# A High-Efficiency Spectral Method for Two-Dimensional Ocean Acoustic Propagation Calculations

**DOI:** 10.3390/e23091227

**Published:** 2021-09-18

**Authors:** Xian Ma, Yongxian Wang, Xiaoqian Zhu, Wei Liu, Wenbin Xiao, Qiang Lan

**Affiliations:** College of Meteorology and Oceanography, National University of Defense Technology, Changsha 410073, China; maxianxy@163.com (X.M.); liuwei@nudt.edu.cn (W.L.); xiaowenbin@nudt.edu.cn (W.X.); lanqiang@nudt.edu.cn (Q.L.)

**Keywords:** ocean acoustic propagation, two-dimensional Helmholtz equation, Chebyshev–Galerkin spectral method, Chebyshev collocation spectral method, serial and parallel optimization

## Abstract

The accuracy and efficiency of sound field calculations highly concern issues of hydroacoustics. Recently, one-dimensional spectral methods have shown high-precision characteristics when solving the sound field but can solve only simplified models of underwater acoustic propagation, thus their application range is small. Therefore, it is necessary to directly calculate the two-dimensional Helmholtz equation of ocean acoustic propagation. Here, we use the Chebyshev–Galerkin and Chebyshev collocation methods to solve the two-dimensional Helmholtz model equation. Then, the Chebyshev collocation method is used to model ocean acoustic propagation because, unlike the Galerkin method, the collocation method does not need stringent boundary conditions. Compared with the mature Kraken program, the Chebyshev collocation method exhibits a higher numerical accuracy. However, the shortcoming of the collocation method is that the computational efficiency cannot satisfy the requirements of real-time applications due to the large number of calculations. Then, we implemented the parallel code of the collocation method, which could effectively improve calculation effectiveness.

## 1. Introduction

In recent years, underwater acoustic technology has been widely used to measure ocean characteristics [1], detect underwater targets [2], and implement wireless underwater communication systems [3]. Although the propagation of acoustic waves in the ocean is affected by both seawater and the ocean surface, acoustic waves of a fixed frequency can still be obtained after adding boundary conditions to constrain the elliptic partial differential Helmholtz equation [4]. Recently, our research group used the one-dimensional spectral method to correctly solve the normal modes in underwater sound propagation [5] and atmospheric acoustics [6], demonstrating that the spectral method has the advantages of fast convergence and high accuracy when solving the sound field. However, there are still some problems in this calculation process. For example, the aim of this approach is to solve the simplified parabolic model instead of the original Helmholtz equation, which narrows the scope of application and introduces model error. Currently, many simplified models of the Helmholtz equation have been developed, such as the parabolic equation model, normal mode model, and ray model. Each simplified model has its own applicable conditions; for instance, the parabolic equation model is suitable only for situations in which the physical parameters change slowly with the horizontal distance [7], while the normal mode model is appropriate only for cases in which the physical parameters are constant [8], and the ray model needs to be applied under high-frequency situations [9]. However, actual sound fields are relatively complex; hence, relatively few sound fields meet the constrained requirements of various models, making it difficult to calculate the ocean acoustic propagation through a simplified model. Therefore, it is necessary to develop a direct solution to the two-dimensional Helmholtz equation of acoustic propagation without using simplified models.

Finite difference, finite element, and spectral methods are numerical discretization techniques that are commonly used to solve differential equations [10]. In particular, finite difference methods are extensively utilized [11] and some mature ocean acoustic calculation programs, such as RAM [12] based on the parabolic equation model and Kraken [13] based on the normal mode model, have been developed using finite difference methods. However, the accuracies of finite difference and finite element methods are limited by their format. Compared with the other two methods, spectral methods can obtain high-precision results with a low degree of freedom [14].

The name of a spectral method takes the form of “A–B”, where A is the type of trial function and B is the classification of the test function. We expand the solution under a set of trial functions, select a set of test functions, approximate the solutions as inner products, and finally force the residual to zero. Spectral methods are different from other numerical calculation techniques mainly because globally smooth function clusters are selected as the trial functions and test functions. Chebyshev, Legendre, and Fourier functions are commonly used trial functions, while the test function clusters are divided mainly into Galerkin, Tau, and collocation types, among which the Galerkin and collocation types require the trial function to meet the boundary conditions. Spectral methods have been used in mathematical and atmospheric circulation problems since the 1980s [15,16]. The use of spectral methods in the calculation of ocean acoustic propagation began in 1993. Dzieciuch wrote the first code for the normal mode model approximated by Chebyshev polynomials [17]. In 2019, Colbrook et al. used the boundary-based collocation spectral method to solve the two-dimensional acoustic scattering problem [18]. In the same year, Wise et al. used the Fourier spectral collocation method to solve the distribution of sound sources. Compared with the analytical solution, it verified that the spectral collocation method has fast convergence speed and high accuracy [19]. Evans et al. proposed a Legendre–Galerkin spectral method to solve the differential eigenvalue problem of complex and discontinuous coefficients in ocean acoustics [20]. In recent years, many scholars have studied the direct solutions of two-dimensional partial differential equations using spectral methods. In 1995, Shen used the Chebyshev–Galerkin spectral method to directly solve the two-dimensional Helmholtz equation with Dirichlet and Neumann boundary conditions, and achieved good results [21]. In 2009, Wang et al. used the Chebyshev–Tau and Chebyshev–Galerkin spectral methods to solve the two-dimensional Poisson equation [22]. In 2015, Zhang used the Chebyshev collocation spectral method to successfully solve the Poisson equation and heat conduction equation [23]. In 2019, Sahuck used the Chebyshev–Galerkin spectral method to solve the partial equations with the Dirichlet boundary condition [24]. Thus, we tried to use the spectral method to solve the two-dimensional ocean acoustic propagation.

However, the spectral method solution process is hindered by long computation times. With the rapid development of high-performance computing (HPC) technology, HPC platforms have provided a new way to study the optimization and parallel computing of ocean acoustic propagation. In 2009, Fan and Da conducted parallel calculations of the FOR3D model in a weak three-dimensional situation [25]. In 2013, Fan et al. used MPI and OpenMP to improve the real-time performance of the underwater acoustic detection system [26]. In 2017, Xu and Wang studied the parallel calculation of the FOR3D model on the Tianhe-2 HPC platform [27]. In 2019, Xiao et al. used OpenMP to accelerate and optimize the three-dimensional wedge-shaped seabed model in parallel on an HPC platform. In 2021, Zhu et al. proposed parallelization and optimization to efficiently simulate a three-dimensional underwater acoustic propagation model on the Tianhe-2 supercomputer [28].

In this paper, we first use the Chebyshev–Galerkin and Chebyshev collocation spectral methods to correctly solve the two-dimensional Helmholtz equation with Robin boundary conditions. Considering the collocation method does not need boundary conditions as strict as for the Galerkin methods, we use the collocation method to solve the Helmholtz equation of two-dimensional ocean acoustic propagation. Comparing the numerical calculation results of the collocation method with those of Kraken reveals that the calculation accuracy of the collocation method is higher than that of Kraken. Then, we implement a parallel fortran code for solving two-dimensional partial differential equations using the spectral method, which effectively improves the speed of the program, greatly reducing the running time. Therefore, it is feasible to use the collocation method to directly solve two-dimensional ocean acoustic propagation problems. Furthermore, since spectral methods do not utilize simplified models, there are no model restrictions on the application conditions and the model does not introduce approximation errors. Hence, the proposed method has the advantages of a high calculation accuracy, a short computation time, and a wide application range.

The remainder of this paper is composed as follows. In Section 1, we introduce the current situation and the main problems of ocean acoustic field solutions. In Section 2, we apply the Chebyshev–Galerkin and Chebyshev collocation spectral methods to solve the Helmholtz equation, and then implement a parallel code for the method. We apply our spectral methods to the Helmholtz model equation and ocean acoustic program examples to verify the high accuracy and efficiency of the method in Section 3. Section 4 provides a summary of this research and the corresponding prospects of the approach presented herein.

## 2. Mathematical Process of Spectral Methods and Optimization of the Calculation Program

In this section, we mainly study how to use the Chebyshev–Galerkin and Chebyshev collocation spectral methods to directly solve the two-dimensional Helmholtz equation with Robin boundary conditions. Then, we use some serial and parallel optimization strategies to accelerate the program due to the large computational cost. The two-dimensional Helmholtz equation can be written as:

(1) $$\begin{array}{cc} \; & \frac{\partial^{2}u}{\partial x^{2}}+\frac{\partial^{2}u}{\partial y^{2}}+\alpha \frac{\partial u}{\partial x}+\beta \frac{\partial u}{\partial y}+\gamma u=f,\;\;-1\leq \;x,y\leq 1\\ \; & {\left.\;\left(u+k_{1}\frac{\partial u}{\partial x}\right)\right|}_{\left|x\right|=1}={\left.\;\left(u+k_{2}\frac{\partial u}{\partial y}\right)\right|}_{\left|y\right|=1}=0, \end{array}$$

where u(x,y) is the quantity to be solved and the other variables are known. When solving the above partial differential equations, it is first necessary to discretize the equations to form a linear equation group that can be solved by mature numerical calculation methods. Here, we used the Chebyshev spectral method to discretize the original equation. The continuous and smooth functions u(x,y) and f(x,y) defined in the interval [−1,+1] in Equation (1) can be approximated by the linear combination of the orthogonal function cluster $\phi_{k,l}\left(x,y\right)$:

(2) $$u\left(x,y\right)\approx \;\sum_{k=0}^{N}\sum_{l=0}^{N}{{\hat{u}}_{k,l}\phi }_{k,l}\left(x,y\right)$$

(3) $$f\left(x,y\right)\approx \;\sum_{k=0}^{N}\sum_{l=0}^{N}{{\hat{f}}_{k,l}\phi }_{k,l}\left(x,y\right),$$

where ${\hat{u}}_{k,l}$ and ${\hat{f}}_{k,l}$ are coefficients of $u\left(x,y\right)$ and $f\left(x,y\right)$, respectively, and the function cluster $\phi_{k,l}\left(x,y\right)$ is a two-dimensional trial function. $\phi_{k,l}\left(x,y\right)$ can be expressed as the product of two one-dimensional trial functions:

(4) $$\phi_{k,l}\left(x,y\right)=\phi_{k}\left(x\right)\phi_{l}\left(y\right).$$

In the Chebyshev–Galerkin and Chebyshev collocation spectral methods, the two one-dimensional trial functions are both Chebyshev polynomials or their linear combinations.

### 2.1. Definition and Properties of a One-Dimensional Chebyshev Polynomial

The Chebyshev polynomials $T_{k}\left(x\right)$ defined in the interval [−1,+1] are:

(5) $$T_{k}\left(x\right)=\cos\left(k\cos^{-1}(x)\right)\;\;\;\;\;k=0,1,2\ldots \;$$

**Definition** **1** (inner product and orthogonality)**.** *The Chebyshev polynomials $\left\{T_{k}\left(x\right)\right\}$ satisfies the following (weighted) orthogonal relationship after introducing the following weighted inner product definition:*

(6) $${\langle T_{m},T_{n}\rangle }_{\omega }:=\int_{-1}^{+1}T_{m}\left(x\right)T_{n}\left(x\right)w\left(x\right)dx=\left\{\begin{array}{cc} 0, & m\neq n\\ \frac{\pi }{2}c_{m}, & m=n \end{array}\right.$$

*where the weight function $w\left(x\right)$ is:*

(7) $$w\left(x\right)=\frac{1}{\sqrt{1-x^{2}}}$$

*and the coefficient $c_{k}$ is defined as:*

(8) $$c_{k}=\left\{\begin{array}{cc} 2, & k=0\\ 1, & k>0 \end{array}\right..$$

*For convenience, if the polynomials are, at most, of N-th degree, Equation (6) can be rewritten in matrix form as:*

(9) $$K=\mathrm{diag}\left\{k_{ii}\right\},\;k_{ii}:=\frac{\pi }{2}c_{k},$$

*where the matrix K is a diagonal matrix with dimensions of $\left(N+1\right)\times \left(N+1\right)$ and the diagonal element value is determined by $c_{k}$ in Equation *(8)*.*

**Definition** **2** (Chebyshev transform)**.** *The continuous smooth function $v\left(x\right)$ defined in the interval [−1,+1] can be approximately expanded with $T_{k}\left(x\right)$ as follows:*

(10) $$v\left(x\right)\approx \sum_{k=0}^{N}{{\hat{v}}_{k}T}_{k}\left(x\right),$$

*where N is the truncation order of the spectral method and ${\hat{v}}_{k}$ is the Chebyshev expansion coefficient of function v. The expansion coefficient is:*

(11) $$\begin{array}{cc} \; & \;{\hat{v}}_{k}=\frac{1}{d_{k}}\int_{-1}^{+1}v\left(x\right)T_{k}\left(x\right)w\left(x\right)dx,\;\;\;\;\\ \; & \;d_{k}=\;\int_{-1}^{+1}\frac{T_{k}^{2}\left(x\right)}{\sqrt{1-x^{2}}}dx=\left\{\begin{array}{c} \pi ,\;\;k=0\\ \frac{\pi }{2},\;\;k>0. \end{array}\right. \end{array}$$

*Equations *(10)* and *(11)* are called the inverse and forward Chebyshev transforms, respectively. If the effect of the truncation order is not taken into account, a one-to-one correspondence can be established between the original function $v\left(x\right)$ and its Chebyshev expansion coefficient $v_{k}$ using this pair of transformations, and they are located in the original physical space and spectral space.*

**Definition** **3** (numerical integration method to calculate the spectral expansion coefficient)**.** *In the forward Chebyshev transform expressed as Equation *(11)*, the Gauss–Lobatto integral is usually used to calculate the weighted integral formula:*

(12) $${\hat{v}}_{k}\approx \frac{1}{d_{k}}\sum_{i=0}^{N}v\left(x_{i}\right)T_{k}\left(x_{i}\right)w_{i},$$

*where N is the number of integration nodes, which is taken as the truncation order of the spectral method. The integration nodes and weights are defined as:*

(13) $$x_{0}=1,x_{N}=-1,x_{i}=\cos\left(\frac{\pi i}{N}\right),w_{0}=w_{N}=\frac{\pi }{2N},w_{i}=\frac{\pi }{N}.$$

**Definition** **4** (spectral coefficient of the derivative function)**.** *For any continuous smooth function $v\left(x\right)$ whose domain is [−1,+1], if the first derivative $v^{\prime }\left(x\right)$ of $v\left(x\right)$ is still a smooth function, $v^{\prime }\left(x\right)$ can also be expanded with $T_{k}\left(x\right)$ as:*

(14) $$v^{\prime }\left(x\right)\approx \sum_{k=0}^{N}{{\hat{v}}_{k}^{{}^{\prime }}T}_{k}\left(x\right).$$

*It can be proven that the expansion coefficient ${\hat{v}}_{k}^{\prime }$ of the first derivative of $v\left(x\right)$ and the expansion coefficient ${\hat{v}}_{k}$ of the original function satisfy the following relationship [29]:*

(15) $${\hat{v}}_{k}^{{}^{\prime }}\approx \frac{2}{c_{k}}\sum_{\begin{array}{c} p=k+1\\ p+k=odd \end{array}}^{N}p{\hat{v}}_{p},\;\;\;k\geq 0,$$

*where $c_{k}$ is defined in Equation *(8)*. Equation *(15)* can then be rewritten in matrix form:*

(16) $${\hat{v}}^{\prime }=D\hat{v},$$

*where ${\hat{v}}^{\prime }$ and $\hat{v}$ are $1*\left(N+1\right)$ arrays defined as ${\hat{v}}^{\prime }=\left[{\hat{v}}_{0}^{\prime },{\hat{v}}_{1}^{\prime }\ldots {\hat{v}}_{N}^{\prime }\right]$ and $\hat{v}=\left[{\hat{v}}_{0},{\hat{v}}_{1}\ldots {\hat{v}}_{N}\right]$, respectively. The matrix ***D*** is an upper triangular square matrix with dimensions of $\left(N+1\right)\times \left(N+1\right)$. Similarly, the expansion coefficient ${\hat{v}}^{\prime \prime }$ of the second derivative $v^{\prime \prime }\left(x\right)$ can be written as:*

(17) $${\hat{v}}^{\prime \prime }=D^{2}\hat{v}.$$

### 2.2. Construction and Properties of One-Dimensional Chebyshev–Galerkin Trial Functions

#### 2.2.1. Chebyshev–Galerkin Trial Function Construction

In the one-dimensional Chebyshev–Galerkin spectral method, the construction of the trial function needs to satisfy not only the orthogonality described in Section 2.1 but also the boundary conditions of the equation to be solved. Therefore, in practice, the trial function $\phi_{k}\left(x\right)$ is usually taken as the linear combination of the Chebyshev polynomials $T_{k}\left(x\right)$ and is called the Chebyshev–Galerkin trial function. Its general structure can be expressed as:

(18) $$\phi_{k}\left(x\right)=T_{k}\left(x\right)+a_{k}T_{k+1}\left(x\right)+b_{k}T_{k+2}\left(x\right)\;,\;\;\;k=0,1,2,\ldots ,N-2,$$

where the undetermined combination coefficients $a_{k}$ and $b_{k}$ can be determined by specific boundary conditions. In matrix form, Equation (18) can be rewritten as:

(19) $$\left[\begin{array}{c} \phi_{0}\left(x\right)\\ \phi_{1}\left(x\right)\\ \vdots \\ \phi_{N-2}\left(x\right) \end{array}\right]=S_{x}^{T}\left[\begin{array}{c} T_{0}\left(x\right)\\ T_{1}\left(x\right)\\ \vdots \\ T_{N}\left(x\right) \end{array}\right]$$

(20) $$S_{x}^{T}=\left[\begin{array}{cccccc} 1 & a_{k} & b_{k} & \; & & \\ \; & 1 & a_{k} & b_{k} & \; & \\ \; & \; & \ddots & \ddots & \ddots & \\ \; & \; & & 1 & a_{k} & b_{k} \end{array}\right],$$

where the constant matrix $S_{x}^{T}$ is a rectangular matrix with dimensions of $\left(N-1\right)\times \left(N+1\right)$.

#### 2.2.2. Inner Product and Orthogonality

Similar to the previous section, a series of properties of the Chebyshev–Galerkin trial functions can be obtained. According to the orthogonality satisfied by the Chebyshev polynomials in Equations (6)–(9), the inner product of the Chebyshev–Galerkin trial functions can also be written in matrix form:

(21) $$b_{k,j}={\langle \phi_{j},\phi_{k}\rangle }_{\omega },\;B_{x}=S_{x}^{T}KS_{x},$$

where $S_{x}$ is the transpose of the matrix $S_{x}^{T}$. The inner product of the Chebyshev trial function and Galerkin trial function can be written as:

(22) $$h_{k,j}={\langle T_{j},\phi_{k}\rangle }_{\omega },\;H_{x}=KS_{x},$$

Similarly, the inner product of the Galerkin trial function and its derivative function can be written as:

(23) $$c_{k,j}={\langle \phi_{j}^{\prime },\phi_{k}\rangle }_{\omega },\;C_{x}=S_{x}^{T}KDS_{x},$$

(24) $$a_{k,j}={\langle \phi_{j}^{\prime \prime },\phi_{k}\rangle }_{\omega },\;A_{x}=S_{x}^{T}KD^{2}S_{x},$$

### 2.3. Chebyshev–Galerkin Spectral Method for Solving the Two-Dimensional Helmholtz Equation

We first determined the two-dimensional Chebyshev–Galerkin trial function using Equations (4) and (18), and then discussed how to discretize Equation (1). According to the principle of the method of weighted residuals (MWR), it is necessary to approximate the value of the function *u* to be solved using the Chebyshev–Galerkin trial function for a finite *N*-term expansion and then to make the residual of the equation orthogonal to the space of the test function; that is, the weighted inner products of the residual and each test function are zero. According to Equation (1), we can obtain:

(25) $$\begin{array}{cc} \; & {\langle \frac{\partial^{2}u}{\partial x^{2}},\varphi_{k,l}\rangle }_{\omega }+{\langle \frac{\partial^{2}u}{\partial y^{2}},\varphi_{k,l}\rangle }_{\omega }+\alpha {\langle \frac{\partial u}{\partial x},\varphi_{k,l}\rangle }_{\omega }+\\ \; & \beta {\langle \frac{\partial u}{\partial y},\varphi_{k,l}\rangle }_{\omega }+\gamma {\langle u,\varphi_{k,l}\rangle }_{\omega }={\langle f,\varphi_{k,l}\rangle }_{\omega }, \end{array}$$

where $\varphi_{k,l}$ represents the two-dimensional test function. According to the different methods used in the test function, the spectral method discussed in this paper can be further divided into the Chebyshev–Galerkin spectral method and the Chebyshev collocation spectral method.

#### 2.3.1. Calculate the LHS of Equation (25)

In the Chebyshev–Galerkin spectral method, the test function is taken as the Chebyshev–Galerkin trial function. Therefore, each item in Equation (25) can be expressed in a concise matrix form. For instance, the inner product of the second-derivative term is:

(26) $$\begin{array}{cc} \; & {\langle \frac{\partial^{2}u}{\partial x^{2}},\varphi_{k,l}\rangle }_{\omega }={\langle \sum_{m=0}^{N}\sum_{n=0}^{N}{\hat{u}}_{m,n}\;\phi_{m}^{{}^{\prime \prime }}\left(x\right)\phi_{n}\left(y\right),\phi_{k}\left(x\right)\phi_{l}\left(y\right)\rangle }_{\omega }\\ \; & =\sum_{m=0}^{N}\sum_{n=0}^{N}{\hat{u}}_{m,n}\;{\;\int \int \;}_{-1}^{+1}\phi_{m}^{{}^{\prime \prime }}\left(x\right){\phi_{k}\left(x\right){w\left(x\right)\phi }_{n}\left(y\right)\phi }_{l}\left(y\right)w\left(y\right)dxdy\\ \; & =\sum_{m=0}^{N}\sum_{n=0}^{N}{\hat{u}}_{m,n}\;{\langle \phi_{m}^{{}^{\prime \prime }}\left(x\right),\phi_{k}\left(x\right)\rangle }_{\omega }\;{\langle {\phi_{n}\left(y\right),\phi }_{l}\left(y\right)\rangle }_{\omega }\;=A_{x}UB_{y}^{T}. \end{array}$$

Similarly:

(27) $${\langle \frac{\partial^{2}u}{\partial y^{2}},\varphi_{k,l}\rangle }_{\omega }=B_{x}UA_{y}^{T}.$$

The inner product of the first-derivative term can be rewritten as:

(28) $$\begin{array}{cc} \; & {\langle \frac{\partial u}{\partial x},\varphi_{k,l}\rangle }_{\omega }={\langle \sum_{m=0}^{N}\sum_{n=0}^{N}{\hat{u}}_{m,n}\;\phi_{m}^{{}^{\prime }}\left(x\right)\phi_{n}\left(y\right),\phi_{k}\left(x\right)\phi_{l}\left(y\right)\rangle }_{\omega }\\ \; & =\sum_{m=0}^{N}\sum_{n=0}^{N}{\hat{u}}_{m,n}\;{\;\int \int \;}_{-1}^{+1}\phi_{m}^{{}^{\prime }}\left(x\right){\phi_{k}\left(x\right){w\left(x\right)\phi }_{n}\left(y\right)\phi }_{l}\left(y\right)w\left(y\right)dxdy\\ \; & =\sum_{m=0}^{N}\sum_{n=0}^{N}{\hat{u}}_{m,n}\;{\langle \phi_{m}^{{}^{\prime }}\left(x\right),\phi_{k}\left(x\right)\rangle }_{\omega }\;{\langle {\phi_{n}\left(y\right),\phi }_{l}\left(y\right)\rangle }_{\omega }\;=A_{x}UB_{y}^{T}. \end{array}$$

Similarly:

(29) $${\langle \frac{\partial u}{\partial y},\varphi_{k,l}\rangle }_{\omega }=B_{x}UC_{y}^{T}.$$

The inner product of the original function term can be rewritten as:

(30) $$\begin{array}{cc} \; & {\langle u,\varphi_{k,l}\rangle }_{\omega }={\langle \sum_{m=0}^{N}\sum_{n=0}^{N}{\hat{u}}_{m,n}\;\phi_{m}\left(x\right)\phi_{n}\left(y\right),\phi_{k}\left(x\right)\phi_{l}\left(y\right)\rangle }_{\omega }\\ \; & =\sum_{m=0}^{N}\sum_{n=0}^{N}{\hat{u}}_{m,n}\;{\;\int \int \;}_{-1}^{+1}\phi_{m}\left(x\right){\phi_{k}\left(x\right){w\left(x\right)\phi }_{n}\left(y\right)\phi }_{l}\left(y\right)w\left(y\right)dxdy\\ \; & =\sum_{m=0}^{N}\sum_{n=0}^{N}{\hat{u}}_{m,n}\;{\langle \phi_{m}\left(x\right),\phi_{k}\left(x\right)\rangle }_{\omega }\;{\langle {\phi_{n}\left(y\right),\phi }_{l}\left(y\right)\rangle }_{\omega }\;=B_{x}UB_{y}^{T}. \end{array}$$

#### 2.3.2. Calculate the RHS of Equation (25)

(31) $$\begin{array}{cc} \; & {\langle f,\varphi_{k,l}\rangle }_{\omega }=\sum_{m=0}^{N+2}\sum_{n=0}^{N+2}{\hat{f}}_{m,n}\;{\langle T_{m}\left(x\right)T_{n}\left(x\right),{\phi_{k}\left(x\right)\phi }_{l}\left(y\right)\rangle }_{\omega }\;\;\\ \; & =\sum_{m=0}^{N+2}\sum_{n=0}^{N+2}{\hat{f}}_{m,n}\;{\langle T_{m}\left(x\right),\phi_{k}\left(x\right)\rangle }_{\omega }\;{\langle {T_{n}\left(y\right),\phi }_{l}\left(y\right)\rangle }_{\omega }\;\;=H_{x}^{T}\hat{f}H_{y}, \end{array}$$

where $\hat{f}$ is the spectral expansion coefficient of the known function *f* under the Chebyshev trial function in Equation (1) and U is the spectral expansion coefficient of the unsolved quantity $u\left(x,y\right)$ under the Galerkin trial function.

#### 2.3.3. Calculate the Helmholtz Equation

Substituting Equations (26)–(31) into Equation (25) gives:

(32) $$A_{x}UB_{y}^{T}+B_{x}UA_{y}^{T}+\alpha C_{x}UB_{y}^{T}+\beta B_{x}UC_{y}^{T}+\gamma B_{x}UB_{y}^{T}=H_{x}^{T}\hat{f}H_{y}.$$

To solve U conveniently, the two-dimensional matrices U and $\hat{f}$ are compressed into one-dimensional vectors by columns, which are denoted as $\bar{u}$ and $\bar{f}$, respectively. Equation (32) can be rewritten as:

(33) $$\left(B_{y}⊗A_{x}+A_{y}⊗B_{x}+\alpha B_{y}⊗C_{x}+\beta C_{y}{⊗B}_{x}+\gamma B_{y}⊗B_{x}\right)\bar{u}=H_{y}^{T}⊗H_{x}^{T}\bar{f},$$

where ⊗ is the Kronecker product. This is a linear equation system for the unknown vector $\bar{u}$ and can be solved by using standard numerical methods to obtain the expansion coefficient $\bar{u}$ of the function $u\left(x,y\right)$. Then, we can obtain the value of the function $u\left(x,y\right)$ according to Equation (2).

### 2.4. Chebyshev Collocation Spectral Method for Solving the Two-Dimensional Helmholtz Equation

#### 2.4.1. Definition of the Test Function and Derivation Matrix

In Equation (25), if the test function cluster is selected as the following special function, it is called the Chebyshev collocation spectral method. Taking the *x* direction as an example, specific (N+1) points ${\left\{x_{j}\right\}}_{j=0}^{N}$ in the solution domain are selected as collocation points and the δ function ($\delta \left(x\right)=0,\left(x\neq 0\right)$) is used as the weight function:

(34) $$w_{j}\left(x\right)=\delta \left(x-x_{j}\right).$$

According to the principle of the MWR, the residual is required to be zero at the $\left(N+1\right)$ collocation points, which means that the differential equation is strictly established at the $\left(N+1\right)$ collocation points.

It is still possible to discretize a system of equations in the spectral space, as discussed in Section 2.2, and then transform them back into the physical space to obtain approximate solutions of the original equations. However, due to the features of the test function, the Chebyshev collocation spectral method can directly form a system of equations in the original physical space without having to resort to utilizing the spectral space. Due to this feature, the collocation-type spectral method can easily address variable coefficients and non-linear problems. We next discuss this method and solution in detail.

The calculation of the collocation-type spectral method is carried out mainly in the grid-point space and the calculation accuracy is related to the selection of the collocation points. Thus, the number of collocation points has an important influence on the accuracy of numerical calculations but the calculation time will be longer as the number of collocation points increases. For convenience, the Gauss–Lobatto node is selected as the grid collocation point and the detailed definition is shown in Equation (13). First, we constructed the Chebyshev derivation matrix. At each discrete point, *x* is used to represent the $\left(N+1\right)$-dimensional vector composed of discrete point coordinates and u is used to represent the $\left(N+1\right)$-dimensional vector composed of the function values at these points. Then, the $\left(N+1\right)\times \left(N+1\right)$ Chebyshev derivation matrix $\tilde{D}$ satisfies:

(35) $$u^{\prime }\left(x\right)=\tilde{D}u.$$

Similarly, the second-order derivation matrix is ${\tilde{D}}^{2}$.

#### 2.4.2. Calculate the Helmholtz Equation

The function *u* in Equation (1) is discretized at the collocation points. Similar to the solution process of the Galerkin method, if the function *u* is compressed into a one-dimensional vector in columns, Equation (1) on the left-hand side can be rewritten as:

(36) $$\frac{\partial^{2}}{\partial x^{2}}\to {\tilde{D}}_{N}^{2}⊗I_{M+1}$$

(37) $$\frac{\partial^{2}}{\partial y^{2}}\to I_{N+1}⊗{\tilde{D}}_{M}^{2}$$

(38) $$\alpha \frac{\partial }{\partial x}\to \alpha {\tilde{D}}_{N}⊗I_{M+1}$$

(39) $$\beta \frac{\partial }{\partial y}\to \beta I_{N+1}⊗{\tilde{D}}_{M}$$

(40) $$\gamma \to \gamma I_{\left(N+1\right)\left(M+1\right)},$$

where the number of discrete points in the *x* direction is $\left(N+1\right)$; that in the *y* direction is $\left(M+1\right)$; and $I_{M}$ is an *M*-order identity matrix. Matrix forms of the boundary conditions $R_{x}$ and $R_{y}$ are as follows:

(41) $$\left.u+k_{1}\frac{\partial u}{\partial x}\to R_{x}u_{\left(N+1\right)\left(M+1\right)}=I_{\left(N+1\right)\left(M+1\right)}+k_{1}\left({\tilde{D}}_{N}⊗I_{M+1}\right)\right.$$

(42) $$\left.u+k_{2}\frac{\partial u}{\partial y}\to R_{y}u_{\left(N+1\right)\left(M+1\right)}=I_{\left(N+1\right)\left(M+1\right)}+k_{2}\left(I_{N+1}⊗{\tilde{D}}_{M}\right).\right.$$

The rows of the matrices $R_{x}$ and $R_{y}$ corresponding to the boundaries $\left|x\right|=1$ and $\left|y\right|=1$ are placed into the corresponding positions in matrix L (Equation (43)).

(43) $$\begin{array}{cc} \; & \left.\frac{\partial^{2}}{\partial x^{2}}+\frac{\partial^{2}}{\partial y^{2}}+\alpha \frac{\partial }{\partial x}+\beta \frac{\partial }{\partial y}+\gamma \to L\right.\\ \; & L={\tilde{D}}_{N}^{2}⊗I_{M+1}+I_{N+1}⊗{\tilde{D}}_{M}^{2}+\alpha {\tilde{D}}_{N}⊗I_{M+1}+\beta I_{N+1}⊗{\tilde{D}}_{M}+\gamma I_{\left(N+1\right)(M+1)} \end{array}$$

The symbol “-” above L indicates the modified matrix operator. Then, Equation (44) is solved to obtain u:

(44) $$\bar{L}u_{\left(N+1\right)\left(M+1\right)\times 1}=\bar{f},$$

where the vector f is a discretized description of f(x,y). $\bar{f}$ is modified as follows: the elements at the boundaries $\left|x\right|=1$ and $\left|y\right|=1$ are taken as zero (according to the boundary conditions of Equation (1)).

### 2.5. A Parallel Code for the Spectral Method

After the above-mentioned rigorous mathematical derivation and analysis, the next step is to write code to realize the calculation. As mentioned in Section 2.4, the collocation method calculation has the characteristics of high precision, but its calculation time will gradually become longer with the increase of the number of collocation points. Thus, it is very important to write well-structured code with fast calculation speed.

First, in the process of writing code, pay attention to the consistency of the computer architecture. For example, we use the fortran language to write code and the data is stored in the order of columns. In the process of reading data, it reads according to the principle of storage to improve the performance of the program. Furthermore, we make full use of the already defined arrays, which could reduce the use of new arrays to reduce memory usage. In addition, the calculation time can be shortened by reducing unnecessary calculations and reducing branch judgment statements.

When a certain step of the program contains a large number of data calculations, in most cases, parallel calculation is the most effective speedup method. The core of the program is to calculate a large system of linear equations, which accounts for about 95.5% of the total execution time because it contains a large number of loops and data calculations. Therefore, the code of solving linear equations is a highly active segment that can be calculated in parallel.

In the process of solving a system of linear equations, the iterative method approximates the exact solution through a finite-step iteration, while the direct method directly solves the solution of the matrix with a higher calculation accuracy. In the process of solving linear equations by the direct method, LU factorization is used to triangulate the matrix. The equation Ax=b is to be solved and the matrix A is decomposed into A=LU, where L is the unit lower triangular matrix and U is the upper triangular matrix.

LU factorization of a matrix means that the row and column elements of the diagonal elements $A_{11}$, $A_{22}$... are transformed in sequence. However, there is no relationship between the row and column elements of each diagonal element in the transformation process, but the subsequent diagonal element rows and columns are dependent on the data. OpenMP is used to perform parallel calculations on the row and column of a specific diagonal element; then, each row and each column are divided into blocks, where the number of elements in each block is *n* (*n* is the number of elements after each row of diagonal elements is divided by the number of threads).

In addition, the process of solving linear equations can be replaced by Intel Math Kernel Library (MKL) to simplify the program structure. Moreover, MKL supports OpenMP, which can be accelerated in parallel by calling OpenMP to increase the calculation speed. Therefore, this paper mainly uses the OpenMP scheme, which provides a shared storage, to accomplish parallel computing.

## 3. Test Results and Analysis

In this section, we first apply the Chebyshev–Galerkin and Chebyshev collocation spectral methods to solve the two-dimensional model Helmholtz equation and then compare both the results and computation times of the two numerical calculations. Since the Chebyshev collocation spectral method has a wide range of applications and does not require strict boundary conditions, this method is applied to solve actual ocean acoustic calculation examples and analytical solutions are used to verify the correctness of the numerical results. Comparing the numerical calculation results of the collocation method with those of Kraken confirms the high calculation accuracy of the collocation method. In order to effectively solve the problem of large amounts of calculation and long running times of the program, we designed the parallel program. The test results show that after parallel optimization, the efficiency of the program is greatly improved. In addition, the truncation order *N* is not required to be the same in both directions; however, to facilitate this test, the *N* values in the two directions are assumed to be the same in the numerical experiments. In the following section, *N* refers to the truncation order in one direction unless otherwise specified.

### 3.1. Two-Dimensional Model Helmholtz Equation

In this section, we take the solution of the following model equation as an example for numerical testing and analysis. The numerical calculation program is written in Fortran code and the test platform is a Dell XPS8930 desktop computer equipped with an Intel i7-8700K CPU and 32 GB of memory.

(45) $$\begin{array}{cc} \; & \frac{\partial^{2}u}{\partial x^{2}}+\frac{\partial^{2}u}{\partial y^{2}}+\frac{\partial u}{\partial x}+\frac{\partial u}{\partial y}+u=f,\;\left(x,y\right)\in \left(-1,1\right)\times \left(-1,1\right)\\ \; & {\left.u-\frac{1}{2\pi }\frac{\partial u}{\partial x}\right|}_{|x|=1}={\left.u-\frac{1}{\pi }\frac{\partial u}{\partial y}\right|}_{\left|y\right|=1}=0\\ \; & f\left(x,y\right)=\left(-5\pi^{2}+1\right)\sin\left(2\pi x+\frac{\pi }{4}\right)\sin\left(\pi y+\frac{\pi }{4}\right)+\pi \sin\left(2\pi x+\frac{\pi }{4}\right)\cos\left(\pi y+\frac{\pi }{4}\right)\\ \; & \;+2\pi \cos\left(2\pi x+\frac{\pi }{4}\right)\sin\left(\pi y+\frac{\pi }{4}\right). \end{array}$$

The exact solution of Equation (45) is $u=\sin\left(2\pi x+\frac{\pi }{4}\right)\sin\left(\pi y+\frac{\pi }{4}\right)$.

**Figure 1:**
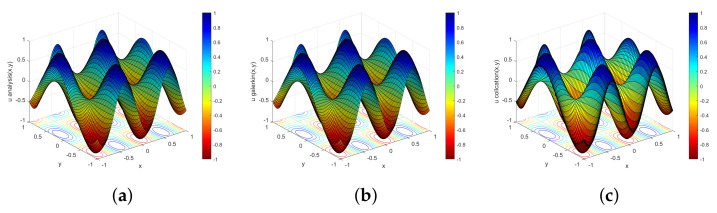
Solution obtained with the analytical method (**a**), Chebyshev–Galerkin spectral method (**b**), and Chebyshev collocation spectral method (**c**).

Equation (45) is solved by the Chebyshev–Galerkin and Chebyshev collocation spectral methods, and the approximate solution obtained are presented in Figure 1. The accuracy is determined by the relative L${}_{2}$-norm of the error between the numerical solution and the exact solution:

(46) $$\mathrm{error}=\frac{{∥u-u_{\mathrm{ana}}∥}_{2}}{{∥u_{\mathrm{ana}}∥}_{2}},$$

where ${∥\cdot ∥}_{2}$ represents the L${}_{2}$-norm of the error, *u* represents the numerical result, and $u_{\mathrm{ana}}$ represents the exact solution.

Figure 1 shows the images of the exact solution and the numerical solutions of the two methods. This function exhibits good smoothness and symmetry but the image is more complex and the gradient changes greatly. It can be clearly seen that the Chebyshev–Galerkin and Chebyshev collocation methods can solve the equation correctly and effectively. Table 1 demonstrates that the two spectral methods show a similar accuracy until N=24, indicating that the approximation accuracy of both numerical methods increases rapidly with increasing truncation order. These two methods can obtain high-precision approximate solutions when the truncation order *N* is small, which verifies the high precision of the spectral method approximations. For almost every increase in the truncation order, the accuracy of the numerical solution increases by an order of magnitude, which verifies the rapid convergence of the spectral methods. The Galerkin method achieves the highest accuracy at N=30 when the error magnitude is $10^{-14}$ and the collocation method achieves the highest accuracy at N=24 when the error magnitude is $10^{-12}$. Although the two spectral methods are both high-precision calculation methods, the collocation method requires a smaller truncation order to achieve the highest accuracy and can effectively reduce the computation time when solving both complex and large-scale problems.

### 3.2. Ocean Acoustic Propagation Example Test

The test in Section 3.1 verifies that the Chebyshev collocation spectral method can directly solve the two-dimensional Helmholtz model equation with high accuracy and fast convergence. Therefore, here, we apply the Chebyshev collocation spectral method to solve the two-dimensional ocean acoustic propagation equation. The collocation method can effectively solve the situation in which the sound speed changes continuously in the solution area. At present, the lower boundary of the solution area is a horizontal layer and the physical quantity changes continuously in each layer. More complex calculation scenarios are still being calculated. Two ocean acoustic propagation examples involving ideal fluid waveguides and spherical waves with analytical solutions are selected to verify the effectiveness of the Chebyshev collocation method in solving the ocean acoustic field.

When the ocean topography and ocean environment parameters are axisymmetric, the circumferential guide number in the cylindrical coordinate system is zero, that is, ∂/∂θ=0. Thus, the Helmholtz equation in cylindrical coordinates can be simplified into a two-dimensional axisymmetric form [30]:

(47) $$\frac{\partial }{\partial r}\left(\frac{1}{\rho }\frac{\partial P}{\partial r}\right)+\frac{1}{r\rho }\frac{\partial P}{\partial r}+\frac{\partial }{\partial z}\left(\frac{1}{\rho }\frac{\partial P}{\partial z}\right)+\frac{k^{2}P}{\rho }=0,$$

where *P* is the sound pressure, ρ is the density, *k* is the wavenumber, *r* is the horizontal axis, and *z* is the vertical axis. The boundary conditions are also required to obtain the specific sound pressure distribution. To simulate the sound field characteristics of a real large-scale sea area, it is often necessary to set the radiation boundary conditions, that is, the conditions that the solution of the wave equation needs to meet at infinity (the sound propagation direction at infinity can be only outward). The Sommerfeld radiation condition expression is:

(48) $$\frac{\partial P}{\partial n}-ikP=0.$$

This condition indicates that on the virtual boundary far from the sound source point, the sound pressure method guide number and the sound pressure itself satisfy Equation (48).

The premise of the radiation boundary conditions is that they correspond to an infinite distance from the sound source point. However, because the calculation area is limited, an “absorption layer” needs to be set at the boundary to avoid false reflections of the sound field by the radiation boundary conditions. The inner side of the absorption layer is connected to the sound field and the radiation boundary condition is set on the outer side. The absorption coefficient of the absorption layer increases rapidly with increasing coordinate values; as a result, the sound pressure rapidly weakens over a short distance. The wavenumber expression in the absorption layer is:

(49) $$\begin{array}{cc} \; & K^{2}=k^{2}\left[1+i\left(\gamma_{r}+\gamma_{z}+\frac{\beta }{27.287527}\right)\right]\\ \; & \gamma_{r}=\left\{\begin{array}{cc} 0, & r\leq L\\ \mu {\left(\frac{r-L}{\Delta L}\right)}^{2}, & r>L \end{array}\right.,\;\gamma_{z}=\left\{\begin{array}{cc} 0, & z\leq H\\ \mu {\left(\frac{z-H}{\Delta H}\right)}^{2}, & z>H \end{array}\right.. \end{array}$$

where the coefficient μ is usually zero; $\gamma_{r}$ represents the change coefficient of the absorption layer in the horizontal direction; $\gamma_{z}$ represents the change coefficient of the absorption layer in the vertical direction; *L* represents the horizontal coordinate of the inner side of the absorption layer in the horizontal direction; and *H* represents the vertical coordinate of the inner side of the absorption layer in the vertical direction. To simulate real ocean conditions, the absorption coefficient β is zero.

To illustrate the calculation results of the sound field, the transmission loss (TL) of the sound pressure is defined as:

(50) $$\mathrm{TL}=-20\log_{10}\left(\frac{\left|P\right|}{P_{0}}\right),$$

where $P_{0}$ refers to the sound pressure 1 m from the sound source. The TL is commonly used to display sound field results and is expressed in units of decibels (dB).

#### 3.2.1. Ideal Fluid Waveguide

**Figure 2:**
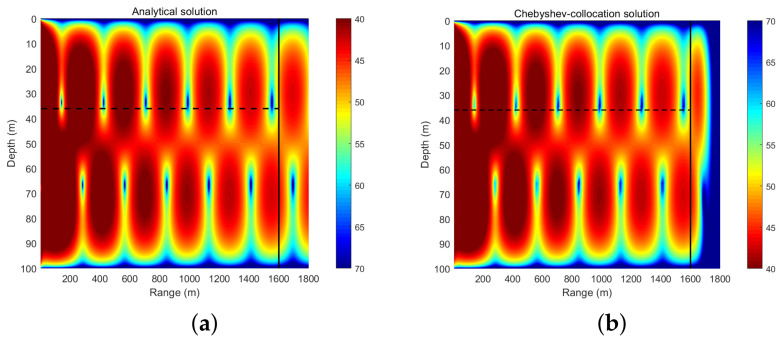
TL field of an ideal fluid wave: the result of the analytical method (**a**) and the result of the collocation method (**b**).

The density of the ocean is uniform at ρ(z)=1 g/cm${}^{3}$, the speed of underwater sound propagation is constant at c=1500 m/s, the sound source is at a depth of 36 m, and the frequency is 20 Hz. The upper and lower boundaries of the solved rectangular area are the pressure release boundary conditions, the left boundary is described by the analytical solution, and the right boundary is described by the radiation boundary condition. The range of the horizontal solution area is 1800 m and the thickness of the absorption layer is 200 m. Therefore, we analyze mainly the calculation area spanning 1600 m in the horizontal direction. A truncation order of N=120 is considered and the results are shown in Figure 2.

**Figure 3:**
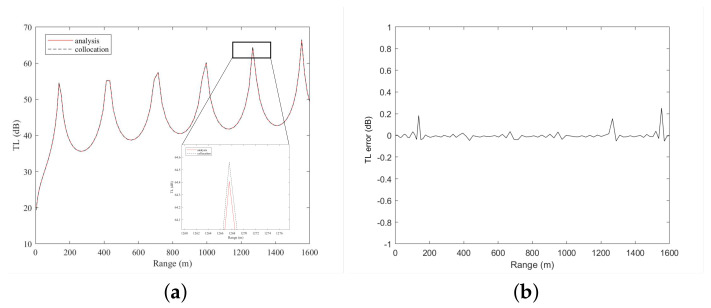
TL curves calculated by the analytical solution (red solid line) and the Chebyshev collocation method (black dashed line) at the sound source depth (**a**) and the error between the numerical solution and the analytical solution (**b**).

Figure 2 reveals that the sound field solution of the non-absorbent layer calculated by the collocation method is very similar to the analytical solution. Figure 3 plots the TL curves of the collocation method and analytical solution at a sound source depth of 36 m. The calculation results of the collocation method and the analytical solution are very similar, but there is a certain error in the sound shadow area. The sound shadow area is relatively complicated and thus the calculation is difficult, which leads to errors in the calculation of the collocation method. The analytical solution is used to analyze the accuracy of the numerical calculation in the non-absorbent layer and the results are shown in Table 2, indicating that the accuracy of the numerical calculation can reach $10^{-4}$ when *N* increases to 80. This example is also solved by the classic Kraken program based on the finite difference algorithm. As shown in Table 2, due to the constraints of the normal wave calculation model and the finite difference discretization method, even if the number of discrete points is increased, the magnitude of the error remains $10^{-1}$, thus the improvement is no longer significant. Table 3 presents the errors of the two numerical calculation methods at a sound source depth of 36 m. The calculation accuracy of the collocation method is similar to that of Kraken when N<120; however, at N=120, the accuracy of the collocation method exceeds that of Kraken by approximately $10^{-4}$. A comparative analysis indicates that the accuracy of the Chebyshev collocation method is higher than that of Kraken, which verifies the high precision of the spectral method. However, the running time of the collocation method is longer than that of Kraken as a result of the large amount of calculations. Thus, we implement the parallel code to accelerate the program.

#### 3.2.2. Spherical Wave

The density of the ocean is uniform at ρ(z)=1 g/cm${}^{3}$, the speed of underwater sound propagation is constant at c=1500 m/s, the sound source is set at the coordinate origin (0, 0), and the frequency is 150 Hz. To avoid singularities in the calculation area, the horizontal solution area is (1, 100) and the vertical solution area is (−50, 50). The analytical solution of the spherical wave is

(51) $$P=\frac{e^{ikR}}{R},\;\;\;R=\sqrt{x^{2}+y^{2}}.$$

**Figure 4:**
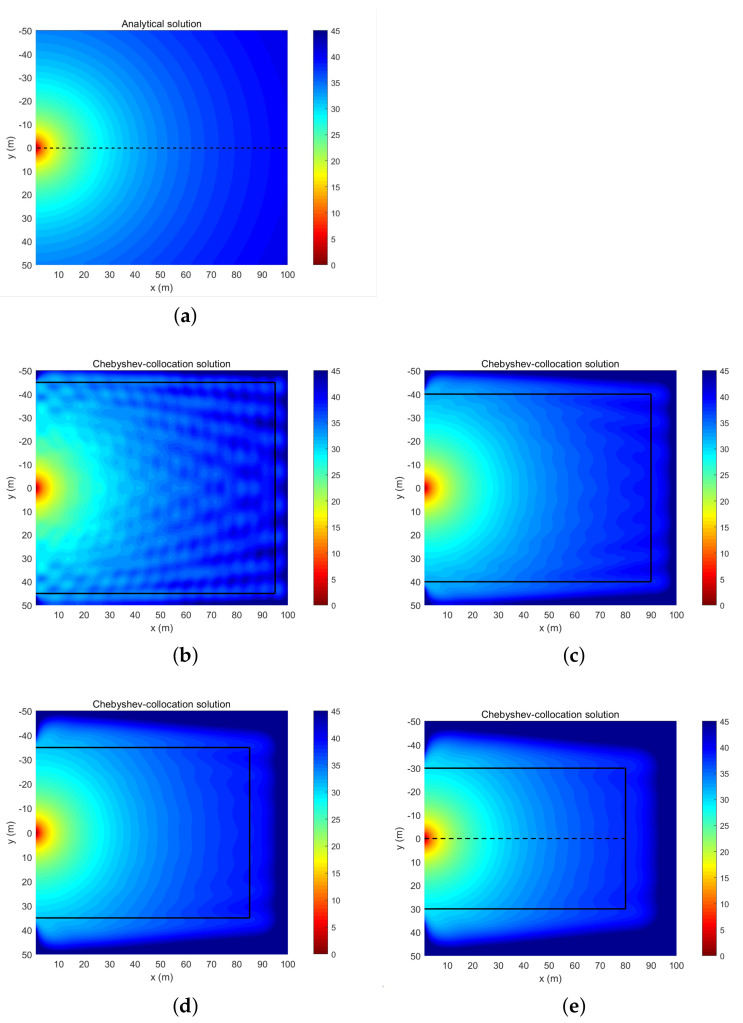
TL field of a spherical wave: the result of the analytical solution (**a**) and the results of the collocation method with the thickness of the absorbing layer being 5 m (**b**), 10 m (**c**), 15 m (**d**), and 20 m (**e**).

The left boundary is described by the analytical solution, whereas the upper, lower, and right boundaries of the solved rectangular area are all assigned radiation boundary conditions, and the thicknesses of the boundary absorption layers affect the calculation accuracy. The truncation order is taken as N=100 and the thickness of the absorbing layer is tested at 5, 10, 15, and 20 m. The calculation results are displayed in Figure 4 and both the errors in the non-absorbent layer area and at the depth of the sound source are shown in Table 4.

**Figure 5:**
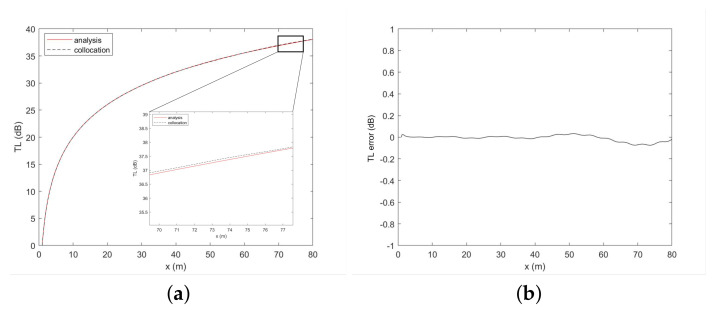
TL curves calculated by the analytical solution (red solid line) and Chebyshev collocation spectral method (black dashed line) at the depth of the sound source (**a**), in addition to the error between the numerical solution and the analytical solution (**b**).

Figure 4 shows that when the absorbing layer is thin, radiation boundary conditions produce false reflections in the sound field, which affect the calculation accuracy. With increasing thickness, the calculation results of the sound field significantly improve. Table 4 further demonstrates that as the absorbing layer thickens, the errors in the entire solution domain and at the sound source depth gradually decrease, while the calculation accuracy increases. Considering the size of the effective computing area and the utilization of computing resources, when the thickness of the absorbing layer reaches 20 m, the numerical calculation accuracy can be ensured and the numerical calculation resources can also be used efficiently. The TL curves at the sound source depth computed by the analytical method (red solid line) are basically identical to those computed by the collocation method (black dashed line) when the thickness of the absorbing layer is 20 m, as shown in Figure 5a. In addition, Figure 5b plots the errors between the numerical solution and the analytical solution. Overall, Figure 5 indicates a slight difference in the calculation results of the collocation method beyond 60 m due to the reflection of the radiation boundary. Therefore, in the following test, we chose the thickness of the absorbing layer to be 20 m.

Table 5 shows the variations in the numerical errors throughout the entire solution domain (except the absorption layer) and at the depth of the sound source with the number of discrete points *N*. With increasing *N*, the calculation error of the Chebyshev collocation spectral method gradually decreases and the numerical calculation accuracy gradually improves. Considering the number of calculations and the required calculation accuracy, we set the truncation order to N=100 for the calculation of spherical waves. However, the computational efficiency of this method cannot meet the requirements of real-time applications because of the excessively large computational load; thus, we utilize a variety of methods to enhance the overall performance in the next section.

### 3.3. A Parallel Code of the Collocation Method

In order to measure the improvement of the program performance by parallel algorithms more effectively, it is necessary to analyze and test on the same high-performance computing platform, namely the Tianhe-2 supercomputer. The configuration of the Tianhe-2 supercomputer is shown in detail in Table 6. For testing, we employed two compilers: gcc version 5.4.0 and Intel mkl-15. In the actual test process, five tests are performed for each optimization and the test time with the shortest time is regarded as the best time. We select the spherical wave for the test, wherein the thickness of the absorbing layer is 20 m and the truncation order *N* is 100 according to the error analysis in Section 3.2.2.

We first write a program with good performance and then start the multi-threaded parallel optimization. The parallel acceleration effect follows the Amdahl speedup law, based on which the parallel effect can be predicted in advance. On a many-core platform, if the maximum number of threads that can be used is *n* and the percentage of the execution time of the parallel part of the program code is *q*, the ideal speedup is calculated as S=1/(1−q+q/n).

**Figure 6:**
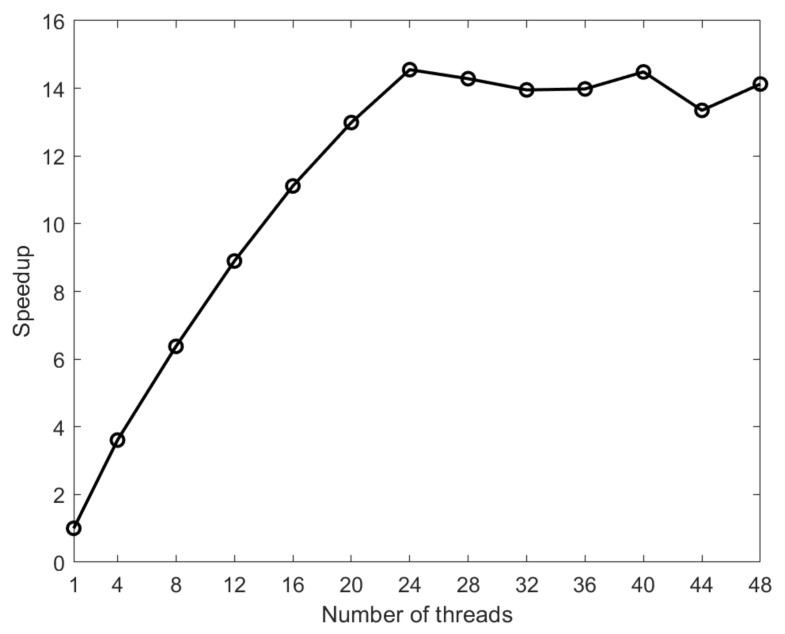
Performance of multithreaded parallelization.

When the same program is executed serially on one node, the speedups present with different numbers of threads, as shown in Figure 6. As the number of threads increases, the computation time of the program (i.e., the running time) decreases dramatically. As shown in Figure 6, the total running time speedup is 14.55 when the number of threads is 24. However, when the number of threads exceeds 24, the speedup decreases; this is because multiple threads share the same processor core in this case and thus competition for resources causes the parallel speedup to diminish. In addition, the forking and joining of operations of too many threads also increase the computation time. When the number of threads is 24, the ideal speedup of the entire program is 19.19 and the actual parallel speedup is 14.55, which does not reach the ideal value. The parallel efficiency is not ideally likely because the system overhead and proportion of non-parallel regions of code both increase when more threads are employed. In comparing the calculation results before and after optimization, the maximum error at all collocation points in the entire calculation area is $10^{-6}$, which shows that optimizing the program does not affect the calculation accuracy.

## 4. Conclusions and Outlook

This paper uses the Chebyshev–Galerkin and Chebyshev collocation spectral methods to directly solve the two-dimensional Helmholtz model equation and uses an analytical solution to verify the correctness of the two methods. The Chebyshev–Galerkin spectral method first constructs a trial function that satisfies the boundary conditions; then, it implements interpolation at the Gauss–Lobatto node; forms a system of linear equations in the spectral space; and finally obtains the original function. However, the two-dimensional ocean acoustic propagation Helmholtz equation is relatively complicated due to the variable boundary conditions and the trial function is difficult to construct. Therefore, realistic ocean acoustic examples are solved by the more widely applicable Chebyshev collocation spectral method. The Chebyshev collocation spectral method approximates the function value at the Gauss–Lobatto node, interpolates physical quantities such as the boundary conditions and sound velocity to the Gauss–Lobatto node, and then directly solves the system of linear equations in the physical space to obtain the original function. The analytical solution is used to verify the correctness of the Chebyshev collocation method and compared with the classic Kraken program, the Chebyshev collocation method has a higher calculation accuracy. Hence, the Chebyshev collocation method can be used to directly solve the two-dimensional underwater acoustic propagation Helmholtz equation without being restricted or constrained by other simplified model application conditions and does not introduce model errors. The Chebyshev collocation method has a wide range of applications and provides results with a high accuracy, which is of great significance in the calculation of realistic ocean sound fields. However, in the process of calculation, the collocation method requires a long running time due to the large amounts of calculation. Therefore, we try to use a parallel code to accelerate the efficiency of the code and achieve a good performance.

This paper is an exploration of solving the two-dimensional Helmholtz equation and mainly introduces the use of the Chebyshev collocation spectral method to solve relatively simple sound propagation problems in ocean media. The calculation of more complicated sound propagation examples in the actual ocean is the key issue of future research. 

## Figures and Tables

**Table 1:** Errors of the results calculated by the Chebyshev–Galerkin and Chebyshev collocation spectral methods varying with the truncation order *N*.

*N*	Galerkin Error	Collocation Error
8	$1.19\times 10^{-5}$	$5.60\times 10^{-2}$
16	$2.73\times 10^{-6}$	$2.68\times 10^{-6}$
22	$5.62\times 10^{-11}$	$6.24\times 10^{-11}$
24	$1.04\times 10^{-12}$	$2.84\times 10^{-12}$
30	$2.51\times 10^{-14}$	$8.15\times 10^{-12}$
32	$2.57\times 10^{-14}$	$5.51\times 10^{-12}$

**Table 2:** Variations in the numerical calculation errors in the entire solution domain with *N*.

*N*	Chebyshev Collocation	Kraken
70	$2.06853038\times 10^{-1}$	$2.41551098\times 10^{-1}$
80	$5.67631508\times 10^{-4}$	$2.41551093\times 10^{-1}$
100	$7.09835092\times 10^{-4}$	$2.41551112\times 10^{-1}$
120	$4.75639831\times 10^{-4}$	$2.41551129\times 10^{-1}$

**Table 3:** Variations in the numerical calculation errors at the sound source depth with *N*.

*N*	Chebyshev Collocation	Kraken
70	$3.32036066\times 10^{-1}$	$2.06786673\times 10^{-3}$
80	$1.52415237\times 10^{-3}$	$2.06475574\times 10^{-3}$
100	$1.73634299\times 10^{-3}$	$2.06635146\times 10^{-3}$
120	$9.33660302\times 10^{-4}$	$2.06476704\times 10^{-3}$

**Table 4:** Variations in the numerical calculation errors in the entire solution domain and at the sound source depth with the thickness of the absorbing layer.

Thickness of the Absorbing Layer (m)	Error in the Solution Domain	Error at the Sound Source Depth
5	0.020149	0.027756
10	0.006115	0.004448
15	0.002959	0.002507
20	0.002099	0.002080

**Table 5:** Variations in the numerical calculation errors in the entire solution domain and at the depth of the sound source with *N*.

*N*	Error in the Entire Domain	Error at the Depth of the Sound Source
60	$1.32700463\times 10^{-1}$	$1.34443432\times 10^{-1}$
80	$4.73780863\times 10^{-3}$	$4.87139228\times 10^{-3}$
100	$2.09898576\times 10^{-3}$	$2.08030059\times 10^{-3}$
120	$1.64301023\times 10^{-3}$	$1.17537743\times 10^{-3}$
140	$1.54173280\times 10^{-3}$	$9.60149598\times 10^{-4}$
160	$1.48522444\times 10^{-3}$	$9.31331082\times 10^{-4}$

**Table 6:** Configuration of single machine node of the Tianhe-2 Supercomputer.

Item	Configuration
Number of CPUs	2
Number of CPU cores	12 cores/CPU × 2 CPUs
GNU compiler	gfortran (version 5.4.0)
Intel compiler	ifort (version 15.0.1)
